# Trophic Drivers of Organochlorine and PFAS Accumulation in Mediterranean Smooth-Hound Sharks: Insights from Stable Isotopes and Human Health Risk

**DOI:** 10.3390/toxics14010058

**Published:** 2026-01-07

**Authors:** Lorenzo Minoia, Guia Consales, Luigi Dallai, Eduardo Di Marcantonio, Michele Mazzetti, Cecilia Mancusi, Lucia Pierro, Emilio Riginella, Mauro Sinopoli, Massimiliano Bottaro, Letizia Marsili

**Affiliations:** 1 Department of Physical Sciences, Earth and Environment, University of Siena, Via Mattioli 4, 53100 Siena, Italy; lorenzo.minoia@unisi.it (L.M.);; 2 Department of Earth Sciences, “Sapienza” University of Rome, 00185 Rome, Italy; 3 Environmental Protection Agency of the Tuscany Region (ARPAT), Via Marradi 114, 57126 Livorno, Italy; m.mazzetti@arpat.toscana.it (M.M.); c.mancusi@arpat.toscana.it (C.M.);; 4 Department of Integrative Marine Ecology, Stazione Zoologica Anton Dohrn—Italian National Institute of Marine Biology, Ecology and Biotechnology, 80121 Naples, Italy; 5 Department of Integrative Marine Ecology, Sicily Marine Centre, Stazione Zoologica Anton Dohrn—Italian National Institute for Marine Biology, Ecology and Biotechnology, 90149 Palermo, Italy; 6 Department of Integrative Marine Ecology, Genoa Marine Centre, Stazione Zoologica Anton Dohrn—Italian National Institute of Marine Biology, Ecology and Biotechnology, 16126 Genoa, Italy; 7 Triton ETS—Marine Research and Conservation, 00195 Rome, Italy; 8 Centro Interuniversitario per la Ricerca sui Cetacei (CIRCE), Department of Physical, Earth and Environmental Sciences, University of Siena, Strada Laterina 8, 53100 Siena, Italy

**Keywords:** persistent organic pollutants, organochlorine compounds, PFAS, stable isotopes, elasmobranchs, Mediterranean Sea, human consumption

## Abstract

Commercial smooth-hound sharks of the genus Mustelus are commonly landed and consumed in Mediterranean fisheries, raising concerns about potential human exposure to persistent contaminants. This study investigated the occurrence of organochlorine compounds (OCs), including hexachlorobenzene (HCB), dichlorodiphenyltrichloroethane (DDT) and its metabolites, and polychlorinated biphenyls (PCBs), together with per- and polyfluoroalkyl substances (PFAS), in muscle and liver tissues of *Mustelus mustelus* and *Mustelus punctulatus* collected in the waters of the Egadi Archipelago (central Mediterranean Sea). OCs were detected in all analyzed samples, with total PCB concentrations reaching higher values in liver compared to muscle tissues, reflecting tissue-specific accumulation and detoxification processes. PFAS were detected in all analyzed muscle samples (1.10–58.5 ng/g w.w.), with PFOS, PFOA and PFNA generally below current European regulatory thresholds, although isolated exceedances were observed. Stable isotope analysis (δ^13^C and δ^15^N) highlighted differences in trophic ecology between the two species and suggested that feeding habitat and trophic position may influence contaminant exposure patterns, particularly in *M. punctulatus*. The human health risk assessment, conducted as a screening-level evaluation, indicated potential concern associated with PCB concentrations in liver tissue, while risks associated with muscle consumption were generally lower. Overall, the integration of contaminant analysis and stable isotopes provides insights into organismal exposure pathways and supports the use of smooth-hound sharks as sentinels of contaminant presence in Mediterranean coastal ecosystems.

## 1. Introduction

The Mediterranean Sea has geographical and ecological characteristics that make it a unique environment [1,2]. It is recognized as a global hotspot of marine biodiversity, hosting approximately 4–18% of the world’s marine species [1,3,4], including numerous endemic and emblematic taxa [3,5]. At the same time, long-standing urban and industrial development along its coasts and intense maritime traffic make the basin highly vulnerable to anthropogenic pressures affecting both biotic and abiotic compartments [6,7,8].

Elasmobranchs are among the most vulnerable components of Mediterranean marine ecosystems due to life-history traits such as slow growth, long lifespan, late sexual maturity and low fecundity. Accordingly, the Mediterranean Sea is one of the three global regions where Chondrichthyes are highly threatened [9]. Multiple stressors contribute to this vulnerability, including climate change [10,11], noise pollution [12,13], bycatch [14], and contamination by both legacy [15,16], and emerging contaminants [17,18,19,20].

Among these, legacy pollutants such as organochlorine compounds (OCs) remain of particular concern due to their persistence and chemical properties, which facilitate their entry into the food web and promote bioaccumulation and biomagnification in top predators [21]. More recently, attention has expanded to per- and polyfluoroalkyl substances (PFAS), a family of highly persistent chemicals widely used in industrial and consumer products [22]. PFAS are resistant to degradation and can accumulate in marine environments and organisms, potentially contributing to long-term exposure patterns in ways that may overlap with those described for OCs [23]. Experimental and field evidence indicates that PFAS may affect key physiological processes in apex predators, including endocrine, immune and reproductive functions [24,25]. More broadly, OCs and PFAS have been associated with disruption of physiological processes in high-trophic-level species, including marine mammals and elasmobranchs [26,27,28,29], and several of these compounds are recognized as endocrine-disrupting chemicals (EDCs), such as DDT and its metabolites, certain PCBs, HCB and several PFAS [24,25].

Once released into the marine environment, these contaminants may accumulate in tissues and have been linked to adverse effects such as reproductive impairment [18,30], immunosuppression [31,32], and metabolic disorders linked to PFAS exposure [33]. Chemical contamination therefore has relevance not only for marine fauna but also for human health [34], as consumer risk increases with POP and PFAS levels in edible species [35], particularly when contaminants accumulate in higher-trophic predators [34,35,36].

Because elasmobranchs occupy intermediate to high trophic levels [37], they can serve as useful sentinels of contaminant presence in marine ecosystems. Stable isotope analysis provides an established approach for investigating trophic ecology and habitat-related feeding patterns in marine organisms [38].

In particular, δ^13^C and δ^15^N are widely applied tracers [39,40]: δ^13^C can inform on carbon sources and feeding habitats, whereas δ^15^N tends to increase with trophic transfers and is commonly used to infer relative trophic position [41,42]. Isotopic niche metrics further support characterization of trophic interactions and trophic specialization within and among populations [43]. Nonetheless, elasmobranch ecological traits also make them sensitive to environmental change and anthropogenic pressures, including overfishing and pollution [9,35,44,45,46]. Despite growing interest in contaminant exposure of marine predators, few studies have described OC and PFAS levels in Mediterranean elasmobranchs [47,48,49,50,51], and integrated approaches combining legacy and emerging contaminants with trophic proxies remain limited. The genus Mustelus includes demersal sharks widely distributed from tropical to temperate waters. This study focuses on two of the 22 described species: *Mustelus mustelus* (Linnaeus, 1758) and *Mustelus punctulatus* (Risso, 1827), which occur in the Mediterranean Sea and the Atlantic Ocean [52,53,54]. These species are commercially exploited due to the market value of their meat [52,55,56,57] and historical records indicate population declines in the Mediterranean over the 20th century [53,55], consistent with their vulnerability to human-induced pressures as also reported by the IUCN [9,58].

The aim of this study is to provide a detailed assessment of contaminant burdens in *M. mustelus* and *M. punctulatus* from the Strait of Sicily, with three objectives: (1) quantify concentrations of OCs and PFAS in liver and muscle tissue; (2) evaluate whether variability in stable isotope signatures (δ^13^C and δ^15^N), is associated with differences in contaminant burdens as proxies of habitat and trophic related exposure; and (3) assess potential risks associated with human consumption of these species. By integrating chemical analyses with trophic ecology, this work offers new insights into the exposure of smooth-hounds to legacy and emerging pollutants in a key Mediterranean fishing area, and contributes to a broader understanding of the implications for both ecosystem health and consumer safety.

## 2. Materials and Methods

### 2.1. Samples Collection and Sampling Activities

In the framework of the LIFE ELIFE (Elasmobranch Low Impact Fishing Experience) project (“https://www.elifeproject.eu/”; accessed on 28 December 2025), co-funded by the European Union’s LIFE Programme, and of the STEORIMA project funded by the European Union’s EMMF, sampling activities were carried out in the waters surrounding the Marine Protected Area (MPA) of the Egadi Islands, particularly off Favignana Island (37°94′27″ N, 12°33′07″ E).

**Figure 1:**
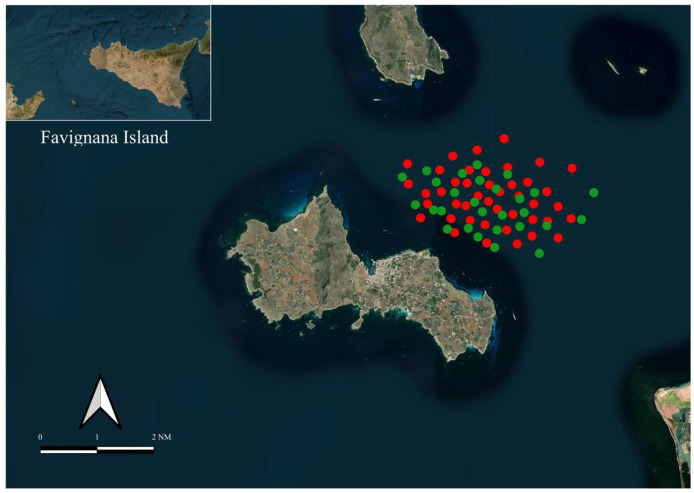
Study area and sampling sites within the Egadi Islands Marine Protected Area (off Favignana Island). Red dots indicate *Mustelus punctulatus*; green dots indicate *Mustelus mustelus*. Map produced with QGIS Desktop 3.10.5 (GRASS 7.8.2).

For the study, three fishing vessels (FVs), ranging from 5.5 to 6.5 m in length, were recruited from Favignana Island. Sampling was conducted during the summer seasons from 2021 to 2023, using trammel nets deployed between 20 and 80 m depth. Following the zonation structure of the MPA, fishing hauls were carried out at two sampling sites located in Zone C, where small-scale fishing is authorized for local FVs under specific regulations (Figure 1).

During the study period, 71 *Mustelus* specimens were collected, including 28 *M. mustelus* and 43 *M. punctulatus*. Taxonomic identification was performed using the dichotomous keys proposed by [59], and morphometric measurements followed [60]. For each specimen, total length (TL, cm), body weight (g), sex, maturity stage, and year of capture were recorded. Maturity staging was based on bibliographic information for each species (size at first maturity); for males, clasper development relative to pelvic fins was also considered.

For chemical analyses, a dorsal muscle sample (5–20 g) was collected from each individual and the entire liver was removed. Samples were stored at −20 °C, wrapped in aluminium foil or placed in glass jars labelled internally and externally, until analysis. Organochlorine compounds were analyzed in all collected specimens, whereas PFAS and stable isotope analyses were conducted on subsets of samples due to funding constraints. Detailed instrumental settings and step-by-step analytical procedures for OCs, PFAS and stable isotope analyses are provided in Supplementary Methods S1–S3.

### 2.2. Organochlorine Contaminant Determination

Determination of HCB, DDTs and PCBs was performed at the Department of Physical Sciences, Earth and Environment, University of Siena, following a modified U.S. Environmental Protection Agency (EPA) 8081/8082 method [28]. Freeze-dried tissues (5–20 g) were solvent-extracted and purified prior to instrumental analysis. Samples were spiked with PCB30 as a surrogate standard [61] and PCB209 (DecaCB) was used as an internal standard for quantification. Quantification of OCs was carried out by high-resolution capillary gas chromatography equipped with an electron capture detector (GC–ECD; Agilent 6890 N with 63Ni ECD). Target analytes included HCB, DDT isomers and metabolites (op’DDT, pp’DDT, op’DDD, pp’DDD, op’DDE, pp’DDE), and 30 PCB congeners. Total PCBs (ΣPCBs) were calculated as the sum of detected congeners, and total DDTs (ΣDDTs) as the sum of the above DDT-related isomers. Limits of detection (LOD) were calculated from replicated blanks (*n* = 20) as mean + 2 SD; censored data were replaced with LOD/2 [62]. Lipid content (EOM%) was determined gravimetrically and water content was calculated from pre- and post-lyophilization weights. Results were expressed as ng/g wet weight (w.w.), and OCs were additionally reported as ng/g lipid weight (l.w.) where relevant.

### 2.3. Per- Polyfluoroalkyl Substances Determination

PFAS were determined in muscle tissue from a subset of the *Mustelus* specimens analyzed for organochlorine compounds. Due to funding constraints, PFAS analyses were performed on 16 randomly selected individuals. Samples were stored at −20 °C until analysis. Chemical analyses were performed at the ARPAT-AVL laboratory using a QuEChERS-based extraction followed by ultra-high-performance liquid chromatography coupled with high-resolution mass spectrometry (UHPLC–HRMS; Thermo Fisher Scientific Orbitrap, Waltham, MS, USA). The analytical procedure is validated and accredited according to UNI EN ISO 17025 for PFOS analysis in whole fish samples [63]. Nine target PFAS compounds were quantified: PFBS, PFHxA, PFOS, PFNA, PFDeA, PFUnA, PFDoA and PFTrDA. Quantification was performed using isotope-labelled internal standards and calibration curves; additional confirmation was obtained using MS/MS acquisition.

### 2.4. Stable Isotopes Analysis (SIA)

Stable isotope analyses (δ^13^C and δ^15^N) were performed on a subset of the Mustelus specimens analyzed for organochlorine compounds. Due to funding constraints, 25 freeze-dried muscle samples were randomly selected for stable isotope analysis, including *M. mustelus* (*n* = 11) and *M. punctulatus* (*n* = 14). Approximately 0.2–0.3 mg of homogenized material was weighed into tin capsules and analyzed by elemental analyzer–isotope ratio mass spectrometry (EA–IRMS) using a Thermo Finnigan FLASH EA 1112 Series CHN Analyzer coupled to a Thermo Finnigan Delta Plus XP isotope ratio mass spectrometer. Isotopic ratios calibrated against international standards (IAEA-CH-6 and IAEA-CH-7; International Atomic Energy Agency, Vienna, Austria) and reported in permill delta notation (δ ‰) relative to the international standard V-PDB for carbon and N_2_ AIR for nitrogen [64]. Analytical error is calculated as cumulative error of duplicates and standards, resulting above 0.3‰ for all tested samples and with average errors of 0.14‰ for Nitrogen and 0.20‰ for Carbon.

### 2.5. Statistical Analyses

All statistical analyses were conducted using STATISTICA 7.1 software unless otherwise specified. Descriptive statistics (mean, standard deviation, minimum and maximum) were calculated to summarize the dataset and describe variability across the measured parameters. Data normality was assessed using the Shapiro–Wilk test, which evaluates the null hypothesis that values are normally distributed (*p* > 0.05). In this study, all investigated groups returned *p*-values below 0.05; therefore, non-parametric statistical methods were applied. Differences among multiple independent groups were tested using the Kruskal–Wallis test, a non-parametric alternative to one-way ANOVA. When the Kruskal–Wallis test indicated significant differences, post-hoc pairwise comparisons were performed using the Kolmogorov–Smirnov test (*p* < 0.10) to examine differences between two independent samples. Exact *p*-values are reported where applicable.

### 2.6. Human Health Risk Assessment

The potential health risks associated with the consumption of shark tissues (muscle and liver) were assessed for both species and evaluated separately for non-carcinogenic and carcinogenic effects. For non-carcinogenic risk, the Hazard Ratio (HR) was calculated by comparing the estimated daily intake (EDI) with the reference dose (RfD) recommended by the U.S. Environmental Protection Agency (EPA):

$$EDI=\frac{C\times DR}{BW}\;\mathrm{and}\;HR=\frac{EDI}{RfD}$$

where C is the concentration of OCs (ng/g w.w.), DR is the daily consumption rate of shark (10.2 g person^−1^ day^−1^), and BW is the average adult body weight (70 kg). The RfD values used were 500 ng/kg/day for DDT [65] and 20 ng/kg/day for PCBs [66]. Carcinogenic risk (CR) associated with OC exposure through fish consumption was estimated following USEPA guidelines using:

CR=EDI×CSF

where CSF is the oral cancer slope factor (3.4 × 10^−7^ and 2 × 10^−6^ (ng/kg/day)^−1^ for DDTs and PCBs, respectively) [66]. Risk values were interpreted as follows: <10^−6^ negligible, 10^−6^–10^−4^ an area of concern, and >10^−4^ unacceptable [67]. The hazard ratio for carcinogenic risk was calculated following [68]:

$$HR=\frac{EDI}{BMC}\;\mathrm{and}\;BMC=\frac{Risk\times BW}{Fish\;consumption\times CSF}$$

where BMC is the benchmark concentration for cancer effects, Risk was set to 10^−6^ for lifetime exposure, and Fish consumption is the consumption rate normalized by body weight (g/kg/day).

## 3. Results and Discussion

The contaminant patterns observed in *M. mustelus* and *M. punctulatus* primarily reflect bioaccumulation processes at the individual level, influenced by age, tissue type, metabolic capacity and exposure history. Although stable isotope data were used to explore potential links between trophic ecology and contaminant levels, the present dataset does not allow direct demonstration of biomagnification, as contaminant concentrations in prey species were not assessed. Consequently, relationships between isotopic signatures and contaminant burdens should be interpreted as indicative of potential trophic exposure pathways rather than evidence of contaminant amplification along the food web.

### 3.1. Biological Parameters

Collected specimens ranged from 44.5 to 150 cm in total length and from 1.96 to 14 kg in weight for *M. mustelus* (*n* = 40), and from 55 to 152 cm in total length and from 6 to 16 kg in weight for *M. punctulatus* (*n* = 43). All individuals were sampled within the Egadi Islands Marine Protected Area, and toxicological analyses were conducted on liver and muscle tissues. Morphometric and biological data, including sex, maturity stage, total length and weight, were recorded for each specimen, allowing a comprehensive characterization of interspecific and individual variability (Table S1).

### 3.2. Organochlorine Compounds Distribution

Analyses conducted on liver and muscle tissues revealed the presence of organochlorine contaminants in both Mustelus species, including hexachlorobenzene (HCB), 30 polychlorinated biphenyl (PCB) congeners, and DDT together with its main metabolites (DDE and DDD), detected in both op’ and pp’ isomeric forms. These compounds were consistently found in all analyzed samples.

Contaminant concentrations were evaluated by species and tissue type (liver and muscle), allowing the characterization of tissue-specific accumulation patterns and interspecific variability. As organochlorine compounds are highly lipophilic, concentrations were expressed on a lipid weight basis to facilitate comparisons among tissues and with previously published data. To summarize the distribution of the measured concentrations, a parametric analysis was applied.

Table 1 reports mean, median, minimum, and maximum values, as well as standard deviations, for HCB, total PCBs and total DDTs, together with diagnostic ratios commonly used to interpret contamination sources and degradation processes.

Higher concentrations of all organochlorine compounds were observed in liver compared to muscle tissues in both species, reflecting tissue-specific accumulation patterns. The liver plays a central role in the uptake, metabolism and storage of lipophilic contaminants, and elevated hepatic concentrations primarily reflect organismal exposure history and physiological processing. In contrast, muscle tissue is more relevant for trophic transfer and human exposure, as it represents the tissue most commonly consumed by predators and humans. Contaminant levels in liver tissues mainly indicate toxicological relevance at the individual level, whereas muscle concentrations are more directly linked to ecological transfer and food safety considerations.

The ratios between DDT isomers, including pp’DDE/pp’DDT, pp’DDE/DDTs and Σop’DDTs/DDTs (Table 1), provide information on the origin and timing of environmental contamination. Elevated pp’DDE/pp’DDT and pp’DDE/DDTs ratios were observed in both species and tissues, suggesting advanced degradation of the parent compound. The Σop’DDTs/DDTs ratio exceeded 0.20 in most samples, with values ranging from 0.25 to 0.29, except in liver tissues of both *M. mustelus* and *M. punctulatus*, where lower values (0.07) were recorded. To further investigate the likely source of contamination, the DDTs/PCBs ratio was calculated, as this index allows discrimination between predominantly agricultural and industrial inputs [69]. In the analyzed samples, DDTs/PCBs ratios ranged from 0.31 to 0.34 in liver tissues and from 0.31 to 0.38 in muscle tissues of both species. These values are indicative of a predominantly industrial origin of contamination, likely associated with urbanized coastal areas, industrial activities, intense maritime traffic and coastal waste disposal sites [70].

The pp’DDE/pp’DDT ratio is commonly used to assess the presence of recent DDT inputs [71]. In commercial DDT formulations, this ratio is approximately 0.05, whereas higher values indicate degradation of the parent compound and the absence of recent applications. In all analyzed samples, pp’DDE/pp’DDT ratios exceeded 3, values consistent with those reported for other Mediterranean Sea studies [51,72], and indicative of historical rather than recent DDT contamination.

The pp’DDE/DDTs ratio was also examined to evaluate the extent of metabolic transformation of DDT. Values equal to or below 0.6 are generally considered critical, as they may indicate recent inputs [73], whereas higher values suggest the absence of recent contamination. In the present study, *M. punctulatus* exhibited values equal to or above this threshold in both liver (0.82) and muscle (0.60). In *M. mustelus*, liver values were above the threshold (0.81), while muscle values were slightly below it (0.57).

Taken together, these ratios consistently indicate the absence of recent DDT inputs and support a scenario of past exposure combined with ongoing metabolic degradation in the analyzed organisms. The Σop’DDTs/DDTs ratio is also informative regarding the type of DDT formulation used. In regulated commercial formulations, op’ isomers typically account for less than 20% of total DDTs, whereas higher proportions (>0.20) are characteristic of technical DDT [74]. In the present study, with the exception of liver tissues in both species (Σop’DDTs/DDTs = 0.07), observed values ranged from 0.23 to 0.47 in liver and from 0.25 to 0.44 in muscle tissues. Technical DDT has been widely used in the production of dicofol, an acaricide and miticide applied in citrus and cotton cultivation. Therefore, the observed isomeric patterns suggest the possible contribution of contamination sources related to this compound [75].

#### PCB and DDT Profiles

Using Aroclor 1260 as the reference standard for PCB characterization, Hexa-chlorinated and Hepta-chlorinated biphenyls emerged as the most abundant congener groups in both Mustelus species and in both analyzed tissues. Together, these congeners accounted for more than 30% of the total PCB burden.

**Figure 2:**
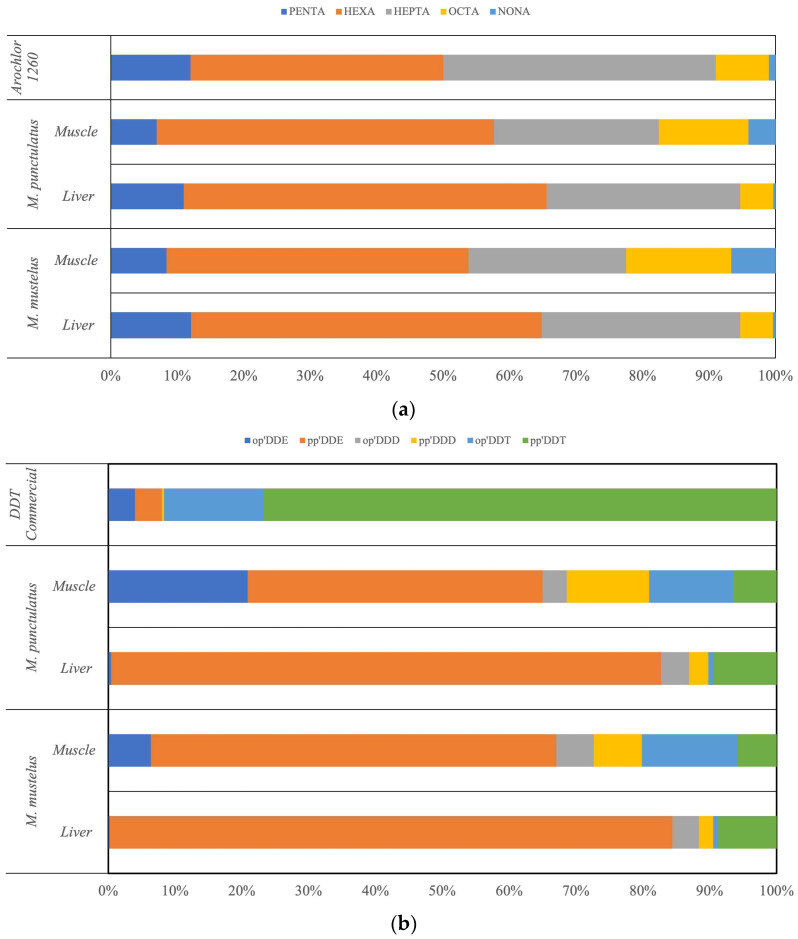
Percentage composition of (**a**) PCBs divided by chlorine content (Penta-CBs, Hexa-CBs, Hepta-CBs, Octa-CBs, Nona-CBs) and (**b**) DDT-related compounds in liver and muscle of *Mustelus mustelus* and *Mustelus punctulatus*.

In muscle tissues of both *M. mustelus* and *M. punctulatus*, the relative abundance of PCB homolog groups followed a consistent pattern: Hexa-CBs (43.27–43.59%) > Hepta-CBs (24.86–26.44%) > Octa-CBs (17.78–18.05%) > Penta-CBs (8.63–8.66%) > Nona-CBs (3.30–5.43%). A similar distribution was observed in liver tissues, although with a higher relative contribution of highly chlorinated congeners: Hexa-CBs (53.93–54.21%) > Hepta-CBs (29.82–30.94%) > Penta-CBs (9.15–10.12%) > Octa-CBs (5.45–5.54%) > Nona-CBs (0.40–0.44%) (Figure 2a). The predominance of highly chlorinated PCB congeners observed in this study is consistent with previous findings from the Mediterranean Sea basin [35,49], indicating a recurring pattern of PCB composition in this geographical area. In particular, congeners such as PCB153, PCB138, PCB187 and PCB180 were frequently detected in both liver and muscle tissues. These compounds have also been widely reported in other marine apex predators, including cetaceans, pelagic fish and sharks [8,28,76,77], reflecting their high chemical stability and resistance to degradation in both environmental matrices and biological tissues [48].

The relative contribution of DDT and its metabolites in liver and muscle tissues of *M. mustelus* and *M. punctulatus* is shown in Figure 2b. Following its release into the environment, DDT undergoes progressive degradation, primarily yielding the metabolites DDE and DDD. Among these, DDE is characterized by greater chemical stability and higher environmental persistence compared to the parent compound.

In the analyzed samples, pp’DDE was the predominant isomer, with proportions substantially exceeding those present in the original technical formulation (approximately 4%). In contrast, pp’DDT, which originally represented the major component of the commercial mixture (77.1%), accounted for only 6.56–8.54% of total DDTs in liver and muscle tissues of *M. mustelus* and for 8.42–8.76% in those of *M. punctulatus*.

### 3.3. PFAS Contamination Levels in Elasmobranch Tissues

Sixteen specimens of *Mustelus* sp. (8 females and 8 males), collected along the coasts of Favignana Island between 2021 and 2023, were analyzed to determine PFAS concentrations in muscle tissue (Table S2). Total length ranged from 46 to 152 cm, and body weight from 0.3 to 16 kg. Biometric parameters were recorded to contextualize chemical concentrations within the biological profiles of the analyzed individuals.

**Figure 3:**
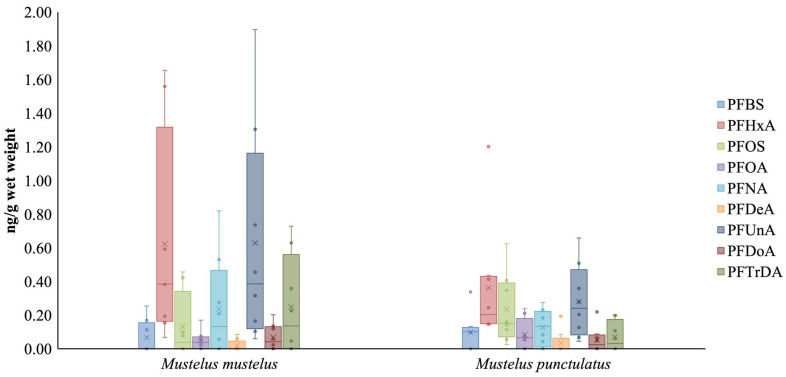
PFAS concentrations (ng/g wet weight) in muscle tissue of *Mustelus* sp. individuals (*n* = 16). collected off Favignana Island.

PFAS concentrations and compositional profiles detected in muscle tissue are reported in Figure 3. All nine target PFAS compounds investigated in this study were detected in every analyzed sample, indicating widespread exposure of *Mustelus* sp. to these substances. Concentrations ranged from 1.10 to 58.5 ng/g wet weight, reflecting variability among individuals.

Several PFAS compounds, including PFHxA, PFOS and PFNA, were detected at variable concentrations, with some values close to or below the analytical limits of quantification. Overall, PFAS concentrations measured in *Mustelus* sp. were lower than those reported for apex predators such as the white shark (*Carcharodon carcharias*) or for some coastal shark species, suggesting comparatively lower exposure or accumulation levels. These differences may be related to ecological and biological factors, including habitat use, feeding habits and body size.

The demersal ecology of *Mustelus* sp. may partly explain the observed PFAS patterns, as pelagic species are often more exposed to diffuse and long-range pollution sources. In this context, Lee [29] reported significantly higher PFAS concentrations in sharks collected from densely populated and industrialized areas, such as the New York Bight, compared to less impacted regions such as the Bahamas. The range of PFAS concentrations observed in the present study is consistent with regional variability driven by local anthropogenic pressure, which may also influence exposure levels in *Mustelus* sp. depending on habitat and sampling location [29].

Previous studies have highlighted species-specific PFAS accumulation patterns in sharks. Mehdi [78] reported predominance of PFOS and long-chain perfluoroalkyl acids, including perfluorotridecanoic acid (PFTrDA), in coastal sharks from the South Atlantic Bight, with particularly high concentrations in bonnethead sharks (*Sphyrna tiburo*). In the present study, PFTrDA was detected in *Mustelus* sp. muscle samples at lower concentrations, generally around 1 ng/g w.w., supporting interspecific variability in PFAS accumulation likely related to differences in ecology, diet and physiological processes [78].

Marciano [79] further demonstrated that PFAS concentrations, particularly long-chain compounds such as PFTrDA, can be substantially higher in blood plasma than in muscle tissue in white sharks. This tissue-specific distribution reflects the strong affinity of certain PFAS for plasma proteins and highlights the importance of tissue selection when comparing PFAS levels across studies and species [79].

The relationship between PFAS concentrations and total body length was examined to explore potential size-related accumulation patterns. No clear correlation between PFAS levels and body length was observed, suggesting that PFAS accumulation in *Mustelus* sp. does not follow a simple size-dependent trend.

Statistical analyses performed on muscle samples from the 16 analyzed individuals, equally divided between males (*n* = 8) and females (*n* = 8), revealed sex-related differences for specific PFAS compounds. The Kruskal–Wallis test identified statistically significant differences between males and females for PFDoA and PFTrDA. These results were further supported by the Kolmogorov–Smirnov test, which confirmed significant differences for the same compounds (*p* < 0.10; Figure S1). With a larger sample size, these differences could be explored in greater detail to better assess potential sex-related variability in PFAS accumulation.

Overall, the detection of PFAS in *Mustelus* sp., although at lower concentrations than those reported for apex predators, confirms the presence and persistence of these contaminants in Mediterranean coastal environments. Given the known bioactive properties of PFAS, their occurrence in shark tissues may have potential ecological and biological implications, particularly in relation to physiological processes such as metabolism, immune function and reproduction, even at sublethal exposure levels. Considering the intermediate trophic position of *Mustelus* sp., PFAS presence likely reflects environmentally driven exposure rather than trophic amplification, supporting the role of this genus as a useful indicator of contamination within coastal food webs. Continued monitoring across species, tissues and trophic levels is therefore warranted to better understand PFAS distribution, exposure pathways and their potential ecological relevance.

### 3.4. Stable Isotopes Analysis (δ^13^C and δ^15^N)

**Figure 4:**
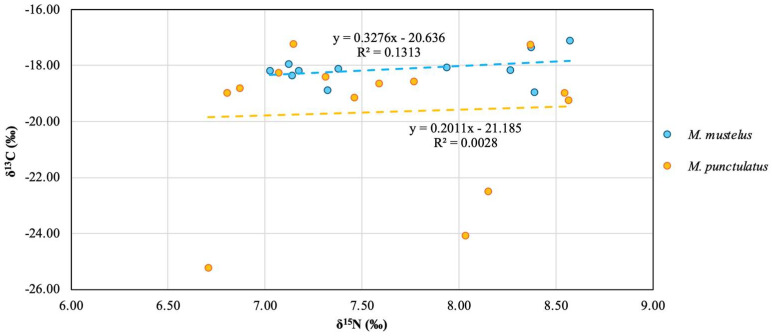
Relationship between the two isotopic signatures δ^13^C (‰) and δ^15^N (‰) of the two species of *Mustelus*.

For the isotopic analyses, 25 samples were used, and the results are shown in Table S3. The isotopic values revealed differences between the two *Mustelus* species. Figure 4 illustrates the relationship between the stable isotopes of carbon (δ^13^C) and nitrogen (δ^15^N) for the two demersal shark species, *M. mustelus* and *M. punctulatus*.

The δ^13^C values observed in this study are generally lower than those reported by Di Lorenzo [54], with most values falling below −18‰. Such differences may reflect variations in feeding ecology and habitat use between the investigated populations. However, δ^13^C values are also influenced by lipid content, which is known to bias carbon isotope ratios toward more negative values. Since lipid extraction was applied in the study by Di Lorenzo [54] but not in the present work, this methodological difference likely contributes to the observed discrepancy and limits direct quantitative comparison between studies.

In this context, δ^13^C values are interpreted here primarily as qualitative indicators of relative habitat-related feeding patterns rather than precise tracers of carbon sources. Lower δ^13^C values have been associated with differences in primary production sources and foraging environments [80,81,82] and are discussed here in relation to relative habitat use rather than as definitive evidence of benthic or pelagic feeding.

Regarding δ^15^N, values were also slightly lower compared to those reported by Di Lorenzo [54]. This difference may reflect variability in trophic position between populations or differences in prey composition and environmental conditions across study areas. Overall, δ^15^N values showed relatively limited variability, suggesting broadly comparable trophic niches between the two species within the investigated area.

**Figure 5:**
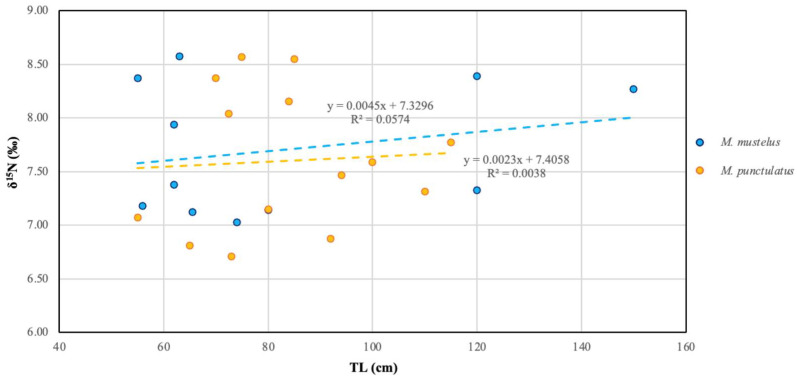
Relationship between total length (TL, cm), and δ^15^N (‰), in *M. mustelus* (blue dots) and *M. punctulatus* (yellow dots).

The relationship between δ^15^N and total body length is shown in Figure 5. In *M. punctulatus*, a weak positive trend was observed; however, the explanatory power of this relationship was very low (R^2^ = 0.0038), substantially lower than that reported by Di Lorenzo [54] (R^2^ = 0.30). This indicates that, in the present dataset, body size explains only a negligible proportion of the observed variability in δ^15^N values, and this relationship should be interpreted in ecological terms with a wider sample size.

Although a slight increase in δ^15^N values with body length was observed in *M. punctulatus*, this pattern remains weak and likely reflects inter-individual variability rather than a clear ontogenetic dietary shift. In *M. mustelus*, no consistent relationship between δ^15^N and body length emerged. Overall, the isotopic data suggest relative stability in feeding strategies throughout ontogeny for both species, with limited evidence for pronounced trophic shifts.

No significant differences in isotopic values were observed between males and females, indicating an absence of detectable sex-related trophic segregation in the analyzed samples. This finding suggests that, for the populations investigated, feeding ecology is broadly similar between sexes, although the limited sample size and natural variability.

#### 3.4.1. Comparative Interpretation of OCs and Stable Isotopes

The analysis of OC levels in relation to body length in *M. mustelus* and *M. punctulatus* revealed differences in accumulation patterns between the two species.

In both species, OC concentrations tended to increase with increasing body length; however, this trend appeared more pronounced in *M. punctulatus* (Figure S2). It is important to note that *M. punctulatus* generally exhibited higher PCB concentrations than *M. mustelus*, although these differences were not consistently statistically significant.

These patterns are consistent with bioaccumulation processes occurring at the individual level and may be influenced by multiple interacting factors, including age, tissue-specific accumulation, ecological traits, and species-specific metabolic capacity. Variability in diet composition, habitat use, and contaminant biotransformation processes may further contribute to the observed interspecific differences. In the absence of contaminant data for prey organisms, these results should be interpreted as indicative of differential exposure and accumulation histories between species.

##### Levels of OCs in Relation to δ^13^C and δ^15^N

The relationship between the carbon isotopic ratio (δ^13^C) and OC concentrations was explored to investigate potential exposure pathways in the two studied species (Figure S3). Positive associations were observed in both species, with a more evident pattern in *M. punctulatus*. Individuals of this species exhibiting higher δ^13^C values tended to show higher OC concentrations

As δ^13^C values provide information on habitat-related feeding patterns and primary production sources, these associations may reflect differences in exposure linked to the environments where feeding occurs. However, δ^13^C should be regarded here as a qualitative indicator of relative habitat use as they do not identify specific contamination sources nor imply direct causal links between feeding habitat and contaminant uptake. The higher OC levels observed in individuals with elevated δ^13^C values may therefore be consistent with increased exposure in certain ecological compartments, rather than with selective consumption of more contaminated prey.

The analysis of the relationship between the nitrogen isotopic ratio (δ^15^N) and OC concentrations revealed positive associations in both species, again more evident in *M. punctulatus* (Figure S4). As δ^15^N values are commonly used as proxies for relative trophic position, these associations may indicate that individuals feeding at slightly higher trophic levels experience greater contaminant exposure.

Overall, the isotopic-contaminant relationships observed suggest that feeding ecology and habitat use may contribute to shaping OC exposure patterns in *M. punctulatus* and *M. mustelus*. Further investigations incorporating direct analyses of contaminant levels in prey organisms would be necessary to clarify the mechanisms leading to contaminant transfer and accumulation within the food web.

#### 3.4.2. PFAS Concentrations in Relation to δ^13^C and δ^15^N

The relationship between PFAS concentrations and δ^13^C was explored to investigate potential associations between PFAS exposure and habitat-related feeding patterns (Figure S5). The data show that higher PFAS concentrations tend to occur in individuals characterized by lower δ^13^C values, suggesting an association with a predominantly benthic diet. This further supports the hypothesis that PFAS accumulation in sharks may stem from exposure to benthic prey, potentially more contaminated than those from pelagic environments. Filter-feeding organisms and other benthic invertebrates, known for their ability to bioaccumulate contaminants from water and sediments, could therefore represent a significant pathway for PFAS transfer within the trophic network. However, future studies incorporating direct analyses of PFAS concentrations in benthic and pelagic prey would be necessary to clarify the role of habitat-related exposure in shaping PFAS patterns in these shark species.

The relationship between PFAS concentrations and δ^15^N values was also examined to explore potential associations with trophic position (Figure S6). However, the available dataset does not allow robust inference in this regard. In *M. mustelus*, the limited number of samples prevents identification of any clear trend, while in *M. punctulatus*, δ^15^N values show relatively limited variability and weak association with PFAS concentrations.

These results indicate that PFAS accumulation in the analyzed species does not follow a clear trophic-level–dependent pattern. Instead, PFAS occurrence appears to be influenced primarily by habitat-related exposure and localized environmental sources.

### 3.5. Human Consumption and Exposure Risk Assessment

The human health risk assessment presented in this study is intended as a screening-level evaluation, based on simplified consumption scenarios and currently available regulatory reference values. Actual dietary exposure may vary depending on local consumption habits, frequency of intake, portion size, and the specific tissues consumed. Quantitative risk metrics were calculated for OCs, whereas PFAS-related considerations are discussed qualitatively in the context of current European regulations and the concentration ranges observed in the analyzed samples.

#### 3.5.1. OCs Evaluation in Exposure Risk Assessment

**Figure 6:**
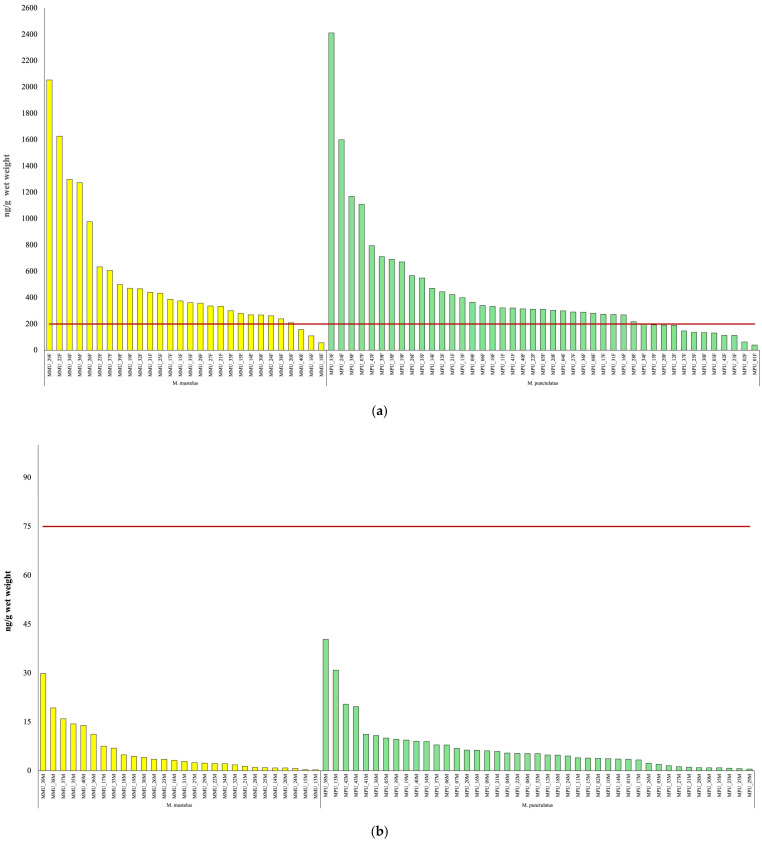
Levels of marker PCB expressed as the sum of four congeners (PCB101, PCB138, PCB153, PCB180) in *M. mustelus* and *M. punctulatus* for liver (**a**) and muscle (**b**). Red line represents the limit threshold according to EU n. 2023/915.

Our values were evaluated with reference to legislative article n°1259/2011 [83] and n°2023/915 [84], which sets the maximum levels for dioxin-like PCBs and non-dioxin-like PCBs in food products. which set maximum levels for dioxin-like PCBs and non-dioxin-like PCBs in food products. According to legislative article n°2023/915, the maximum allowable level for muscle is 75 ng/g wet weight, while for liver and marine organism oils intended for human consumption it is 200 ng/g wet weight. These limits are based on the sum of six PCB congeners (PCB28, PCB52, PCB101, PCB138, PCB153 and PCB180), which represent approximately half of the total non-dioxin-like PCBs in food products and animal feed and are widely used as markers of non-dioxin-like PCB contamination [85]. In the present study, results were calculated using the sum of four of the six congeners included in legislative article n°2023/915 (PCB101, PCB138, PCB153 and PCB180) (Figure 6a,b). Hazard ratio (HR) values were below 1 for both non-carcinogenic and carcinogenic endpoints across all analyzed tissues, except for PCB levels in liver of both species, where HR ranged from 3.91 to 5.72 (Table 2). This indicates that liver consumption deserves specific attention in the context of PCB exposure. Regarding cancer risk (CR), PCB values ranged between 10^−3^ and 10^−5^ in both species and tissues, exceeding commonly used benchmark levels (Table 2).

In contrast, CR values for DDT ranged between 10^−5^ and 10^−7^, suggesting lower carcinogenic concern compared to PCBs. The congeners considered are recognized as useful indicators of exposure to non-dioxin-like PCBs, which may be of concern when present at elevated levels [86].

Demersal species inhabiting coastal and benthic environments may experience sustained exposure to pollutants, as these areas often coincide with nursery and refuge habitats, even when affected by human activities [45]. While pollution is not generally considered the primary threat to sharks compared to overfishing, habitat degradation and climate change, chemical contamination may add pressure to already vulnerable populations [87]. For this reason, contamination levels in shark species that are frequently caught in coastal fisheries and consumed locally warrant specific attention. Organochlorine compounds, including HCB, PCBs and DDTs, can persist and accumulate in marine organisms, potentially contributing to long-term impacts on wildlife and human exposure through diet.

Although the use of OCs has been banned or strictly regulated worldwide, pesticide application and enforcement can remain uneven in some regions, including parts of the Mediterranean area, increasing the likelihood of legacy or unauthorized compounds entering food webs [88]. Along the Mediterranean coast, industrial activities, maritime traffic, slaughterhouses and untreated domestic wastewater represent continuous inputs of pollutants into the marine environment [89,90]. In this context, demersal sharks such as *M. mustelus* and *M. punctulatus* are relevant both ecologically and for food safety, as they are commonly captured by artisanal fisheries and represent a traditional food resource in local communities [52,55,57,91,92]. This raises concerns because contaminants accumulated in shark tissues may be transferred to humans through consumption, with potential long-term effects including carcinogenic risk, endocrine disruption and neurological outcomes [93]. Overall, the risk estimates derived from this study highlight that PCB exposure associated with liver consumption is the main issue emerging from the screening-level assessment and reinforce the need for continued monitoring of PCB marker congeners in commonly consumed elasmobranch products.

#### 3.5.2. PFAS Evaluation in Exposure Risk Assessment

The increasing consumption of shark meat in several regions raises food safety considerations, particularly in light of the widespread occurrence of per- and polyfluoroalkyl substances (PFAS) in marine environments. PFAS can accumulate in marine organisms, including sharks, and seafood consumption represents a relevant pathway for human exposure. In this context, reference values and regulatory limits provide a useful framework to interpret PFAS concentrations measured in edible tissues.

The European Commission’s Regulation (EU) 2023/915 establishes maximum levels for PFAS in fish muscle, including 2 μg/kg w.w. for PFOS, 0.2 μg/kg w.w. for PFOA, 0.5 μg/kg w.w. for PFNA and 0.2 μg/kg w.w. for PFHxS, with an additional limit of 2 μg/kg w.w. for the sum of PFOS, PFOA, PFNA and PFHxS in fish muscle tissue. Although these limits are not specifically defined for sharks, they represent the most appropriate regulatory benchmark currently available for a screening-level interpretation of PFAS concentrations in muscle tissue. In the present study, results were evaluated based on the sum of three of the four PFAS specified in Regulation (EU) 2023/915 (PFOS, PFNA and PFOA) (Table S4).

**Figure 7:**
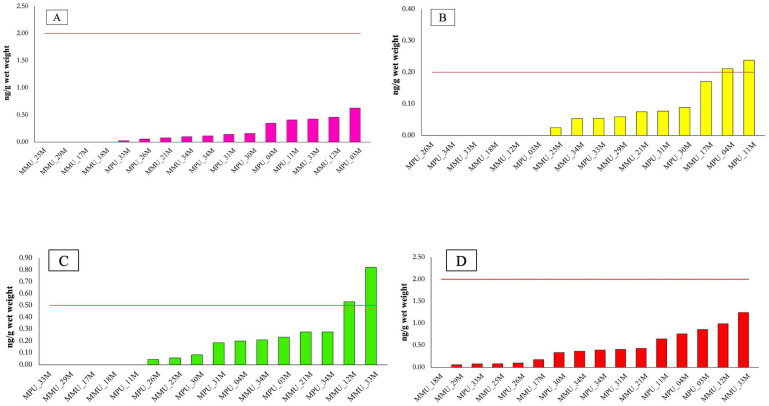
PFAS levels (ng/g wet weight) in muscle tissue of *Mustelus* sp. The red line represents the threshold limit according to EU Regulation No. 2023/915 for PFOS (**A**), PFOA (**B**), PFNA (**C**), and the sum of the three analyzed contaminants (**D**).

PFOS concentrations were within the regulatory limit in all analyzed samples, ranging from below the limit of detection (LOD) to a maximum of 0.636 ng/g w.w. (Figure 7A).

For PFOA, most samples were below the regulatory threshold; however, two samples slightly exceeded the limit, with concentrations of 0.211 ng/g and 0.238 ng/g (Figure 7B). A similar pattern was observed for PFNA: two samples exceeded the maximum allowable concentration (Figure 7C). MMU_12M showed a value just above the limit (0.531 ng/g), while MMU_33M presented a higher concentration (0.821 ng/g), exceeding the threshold.

The sum of PFOS, PFOA and PFNA was also calculated, and no sample exceeded the maximum allowable limit (Figure 7D), indicating that overall PFAS burden, as captured by this partial sum, remained below the regulatory benchmark. Nonetheless, the exceedances observed for individual compounds, particularly PFNA and the MMU_33M sample, indicate that PFAS exposure may be heterogeneous among individuals and may reflect localized environmental conditions or spatial variability in contamination sources. These results suggest that most muscle samples are within current regulatory limits for PFAS, while isolated exceedances highlight the value of continued monitoring in commonly consumed elasmobranch products to better characterize variability and support food safety evaluation.

## 4. Conclusions

This study provides new information on the occurrence and tissue distribution of organochlorine compounds and PFAS in two commercially exploited smooth-hound shark species from the Mediterranean Sea. Overall patterns were consistent with bioaccumulation at the individual level and were shaped by tissue type, interspecific differences, and individual exposure history. Stable isotope analysis contributed to interpreting contaminant variability in relation to habitat and trophic proxy signatures, supporting the identification of plausible exposure pathways rather than demonstrating biomagnification or direct causal links between feeding ecology and contaminant uptake.

From a food safety perspective, the assessment highlighted that PCB concentrations in liver tissues deserve particular attention, whereas exposure estimates associated with muscle consumption were generally lower. PFAS concentrations in muscle were mostly within current European regulatory limits, although isolated exceedances for individual compounds indicate heterogeneous exposure and support the value of continued monitoring in commonly consumed elasmobranch products.

Limitations of the study include subsampling for PFAS and stable isotopes, tissue-specific analyses and the absence of prey data. Despite these constraints, the integrated use of contaminant analysis and stable isotopes represents a valuable approach for assessing organismal exposure and supporting environmental monitoring in Mediterranean coastal ecosystems.

## Figures and Tables

**Table 1:** Levels (ng/g lipid weight) of HCB, PCBs, and DDTs and ratios (DDTs/PCBs, pp’DDE/pp’DDT, pp’DDE/DDTs, Σop’DDTs/ΣDDTs) in *M. mustelus*, *M. punctulatus* tissues collected. Data are reported as mean ± standard deviation (minimum–maximum).

	Tissue	HCB	PCBs	DDTs	$\frac{DDTs}{PCBs}$	$\frac{pp\prime DDE}{pp\prime DDT}$	$\frac{pp\prime DDE}{DDTs}$	$\frac{\Sigma op\prime DDTs}{DDTs}$
*Mustelus* *mustelus*	Liver *n* = 28	13.19 ± 5.24 (2.66–24.84)	3150.16 ± 2280.20 (348.16–9467.97)	946.13 ± 667.69 (149.25–3007.75)	0.32 ± 0.07 (0.21–0.48)	9.73 ± 3.30 (5.40–21.31)	0.81 ± 0.05 (0.67–0.89)	0.07 ± 0.04 (0.02–0.18)
	Muscle *n* = 28	1.75 ± 1.60 (0.28–7.58)	254.94 ± 170.78 (76.15–845.51)	76.50 ± 58.14 (21.61–253.98)	0.31 ± 0.07 (0.08–0.42)	10.70 ± 7.02 (3.47–42.45)	0.57 ± 0.12 (0.23–0.76)	0.29 ± 0.11 (0.13–0.57)
*Mustelus punctulatus*	Liver *n* = 43	11.20 ± 4.34 (2.76–25.21)	2373.74 ± 2047.37 (291.78–9251.41)	726.9 ± 496.26 (82.85–2005.75)	0.34 ± 0.09 (0.18–0.64)	37.61 ± 119.78 (3.09–794.62)	0.82 ± 0.06 (0.59–0.94)	0.07 ± 0.03 (0.02–0.17)
	Muscle *n* = 43	2.04 ± 4.33 (0.32–28.40)	316.20 ± 458.88 (44.97–2633.84)	94.38 ± 102.26 (21.58–615.51)	0.38 ± 0.14 (0.13–0.71)	11.07 ± 11.25 (0.34–73.24)	0.60 ± 0.18 (0.04–0.85)	0.25 ± 0.12 (0.10–0.60)

**Table 2:** The Hazard Ratio (HR) for non-carcinogenic risk, and the carcinogenic risk (CR) for *M. mustelus* and *M. punctulatus* consumed in Italy.

Species	OC Group	Tissue	HR Non-CR	HR CR	CR
*Mustelus mustelus*	PCBs	Muscle	0.0943	0.0785	$3.77\times 10^{-5}$
		Liver	5.7211	4.7637	$2.29\times 10^{-3}$
	DDTs	Muscle	0.0001	0.0004	$1.95\times 10^{-7}$
		Liver	0.0067	0.0237	$1.14\times 10^{-5}$
*Mustelus punctulatus*	PCBs	Muscle	0.1136	0.0946	$4.54\times 10^{-5}$
		Liver	4.7032	3.9162	$1.88\times 10^{-3}$
	DDTs	Muscle	0.0002	0.0006	$2.68\times 10^{-7}$
		Liver	0.0055	0.0193	$9.28\times 10^{-6}$

## Data Availability

The data presented in this study are available on request from the corresponding author.

## Supplementary Materials

The following supporting information can be downloaded at: https://www.mdpi.com/article/10.3390/toxics14010058/s1, Figure S1: Mean PFAS concentrations (ng/g wet weight) in muscle tissue of *Mustelus* sp. (males, n = 8; females, *n* = 8). Bars show mean values and error bars indicate standard deviation; Figure S2: Relationship between total length (cm) and organochlorine compound (OC) concentrations (ng/g lipid weight) in *M. mustelus* and *M. punctulatus*. Concentrations of HCB, ΣDDTs and ΣPCBs are shown as a function of shark length.; Figure S3: Relationship between δ^13^C (‰) and OC concentrations (ng/g lipid weight) in *M. mustelus* and *M. punctulatus*. Concentrations of HCB, ΣDDTs and ΣPCBs are shown as a function of δ^13^C.; Figure S4: Relationship between δ^15^N (‰) and OC concentrations (ng/g lipid weight) in *M. mustelus* and *M. punctulatus*. Concentrations of HCB, ΣDDTs and ΣPCBs are shown as a function of δ^15^N.; Figure S5: Relationship between δ^13^C (‰) and PFAS concentrations (ng/g wet weight) in *M. mustelus* and *M. punctulatus*; Figure S6: Relationship between δ^15^N (‰) and PFAS concentrations (ng/g wet weight) in *M. mustelus* and *M. punctulatus*; Table S1: List of specimens collected in the Egadi Islands Marine Protected Area (MPAEI) during 2021–2023. The table reports sample ID, species, year of capture, sex, maturity stage and total length; Table S2: Biometric and biological information for *Mustelus* sp. individuals analyzed for PFAS. ID: specimen code; TL: total length (cm); W: body weight (kg); F: female; M: male; Table S3: Stable isotope results for *M. mustelus* and *M. punctulatus*. The table includes sample ID, sex, total length (cm) and δ^13^C and δ^15^N values (‰); Table S4: PFAS concentrations measured in muscle samples for PFOS, PFOA and PFNA, together with their sum, reported for comparison with EU Regulation 2023/9155.
