# Supplementary material for: Trophic Drivers of Organochlorine and PFAS Accumulation in Mediterranean Smooth-Hound Sharks: Insights from Stable Isotopes and Human Health Risk
Source: Toxics. 2026 Jan 7;14(1):58. doi: 10.3390/toxics14010058 (PMC12846023; doi:10.3390/toxics14010058)
Supplement: Supplementary file 1 [file toxics-14-00058-s001.zip › toxics-4059521-supplementary.pdf]

Supplementary Material

Trophic Drivers of Organochlorine and PFAS Accumulation in Mediterranean Smooth-Hound Sharks: Insights from Stable Isotopes and Human Health Risk

Lorenzo Minoia[1], Guia Consales[1,*], Luigi Dallai[2], Eduardo Di Marcantonio[2], Michele Mazzetti[3], Cecilia Mancusi[3], Lucia Pierro[3], Emilio Riginella[4,^], Mauro Sinopoli[5], Massimiliano Bottaro [6,7,†], Letizia Marsili [1,8,†]

1 Department of Physical Sciences, Earth and Environment, University of Siena, Via Mattioli 4, 53100 Siena, Italy
2 Department of Earth Sciences, "Sapienza" University of Rome, 00185 Rome, Italy
3 Environmental Protection Agency of the Tuscany Region (ARPAT), Via Marradi 114, 57126 Livorno, Italy
4 Department of Integrative Marine Ecology, Stazione Zoologica Anton Dohrn - Italian National Institute of Marine Biology, Ecology and Biotechnology, Naples, 80121, Italy
5 Department of Integrative Marine Ecology, Sicily Marine Centre, Stazione Zoologica Anton Dohrn - Italian National Institute for Marine Biology, Ecology and Biotechnology, Palermo, 90149, Italy
6 Department of Integrative Marine Ecology, Genoa Marine Centre, Stazione Zoologica Anton Dohrn – Italian National Institute of Marine Biology, Ecology and Biotechnology, Genoa, 16126, Italy
7 Triton ETS - Marine Research and Conservation, Rome, 00195, Italy
8 Centro Interuniversitario per la Ricerca sui Cetacei (CIRCE), Department of Physical, Earth and Environmental Sciences, University of Siena, Strada Laterina 8, 53100 Siena, Italy
^ ER passed away during this study
† These authors contributed equally to this work
* Correspondence: guia.consales@unisi.it

Table of contents

Supplementary Methods S1. Organochlorine contaminant determination (detailed protocol) 2
Supplementary Methods S2. PFAS determination (detailed protocol) 2
Supplementary Methods S3. Stable isotope analysis (detailed protocol) 2
3. Results 3
3.1. Biological parameters 3
3.3. PFAS contamination levels in elasmobranch tissues 5
3.4. Stable Isotopes analysis ($\delta^{13}C$ and $\delta^{15}N$) 6
3.4.1. Comparative interpretation of OCs and Stable Isotopes 7
3.4.1.1. Levels of OCs in relation to $\delta^{13}C$ and $\delta^{15}N$ 8
3.4.2. PFAS concentrations in relation to δ13C and δ15N 9
3.5.2. PFAS evaluation in exposure risk assessment 10

Supplementary Methods S1. Organochlorine contaminant determination (detailed protocol)

Determination of HCB, DDTs and PCBs was performed at the Department of Physical Sciences, Earth and Environment at University of Siena, according to a modified U.S. Environmental Protection Agency (EPA) 8081/8082 method [28]. Samples (5–20 g) were lyophilized using an Edwards freeze drier for 3 days and extracted with n-hexane (PESTINORM, VWR Chemicals) in a Soxhlet apparatus. VWR cellulose thimbles (internal diameter 25 mm, external diameter 27 mm, length 100 mm) were preheated for 30 min at 110°C and pre-extracted for 9 h with n-hexane to remove potential organochlorine contamination.

Each sample was spiked prior to extraction with 2,4,6-trichlorobiphenyl (IUPAC n° 30) as a surrogate compound [61]. After 9 h extraction with n-hexane, the extracts were purified with 95% sulphuric acid (VWR Chemicals) to obtain lipid sedimentation. The apolar fraction was further purified by liquid chromatography on a florisil column dried for 1 h at 110°C. Decachlorobiphenyl (DecaCB – IUPAC n° 209) was used as an internal standard, added prior to extraction and included in the calibration standard (mixture of Aroclor 1260, HCB and pp'- and op'-DDT, DDD and DDE).

Instrumental determination was carried out by high-resolution capillary gas chromatography equipped with an electron capture detector (Agilent 6890 N with 63Ni ECD) and an SBP-5 bonded phase capillary column (30 m, 0.2 mm i.d.). The carrier gas was nitrogen with a head pressure of 15.5 psi (split ratio 50:1). The scavenger gas was argon/methane (95/5) at 40 mL/min. The oven temperature was held at 100°C for 10 min and then increased to 280°C at 5°C/min. Injector and detector temperatures were 200°C and 280°C, respectively. A mixture of specific isomers was used for calibration, recovery evaluation and confirmation of results.

Thirty PCB congeners were quantified (IUPAC n° 95, 99, 101, 118 – penta-CBs; 128, 135, 138, 141, 144, 146, 149, 151, 153, 156 – hexa-CBs; 170, 171, 172, 174, 177, 178, 180, 183, 187 – hepta-CBs; 194, 195, 196, 199, 201, 202 – octa-CBs; 206 – nona-CB). Total PCBs (ΣPCBs) were calculated as the sum of all quantified congeners (representing ~80% of the total PCB peak area). Total DDTs (ΣDDTs) were calculated as the sum of op'DDT, pp'DDT, op'DDD, pp'DDD, op'DDE and pp'DDE.

The limit of detection (LOD) was calculated by analyzing replicated blanks (n = 20) and defined as mean + 2 SD. The LOD for all compounds was 0.1 ng/kg (ppt). For analytical purposes, censored data were replaced with LOD/2 (0.05 ng/kg) [62].

The extracted organic material (EOM%; lipid content) was determined gravimetrically after extraction with n-hexane, and water content (%H2O) was calculated from the weight difference before and after freeze-drying. Results were expressed in ng/g wet weight (w.w.) and, where relevant, in ng/g lipid weight (l.w.).

Supplementary Methods S2. PFAS determination (detailed protocol)

PFAS determination was performed on muscle tissue from a subset of Mustelus specimens previously analyzed for organochlorine compounds; the number of PFAS samples (n = 16) was limited by available funding. Samples were collected from Favignana Island during the summer seasons 2021–2023 and stored at −20°C until analysis.

Chemical analyses were performed at the ARPAT-AVL laboratory following a QuEChERS-based extraction and quantification by ultra-high-performance liquid chromatography coupled with high-resolution mass spectrometry (UHPLC–HRMS; Thermo Fisher Scientific Orbitrap, Waltham, MS, USA). The analytical procedure is validated and accredited in compliance with UNI EN ISO 17025 for PFOS analysis in whole fish samples [63].

A 2 g portion of frozen tissue was placed into a 50 mL glass tube with screw cap to minimize contamination. A 100 µL solution of PFAC-ILS (200 ng/mL) was added and samples were left overnight at −18°C. The following day, a ceramic homogenizer was introduced and 10 mL water and 10 mL acetonitrile were added sequentially. A QuEChERS pouch was added, the tube was shaken vigorously, and samples were centrifuged at 3,500 rpm for 10 min. The supernatant was transferred into a polypropylene tube and stored again at −20°C overnight. The frozen supernatant was then centrifuged at 10,000 rpm for 5 min at −10°C using an SL Plus series centrifuge (Thermo Fisher Scientific, Waltham, MS, USA). Finally, 1 mL of purified supernatant was transferred into a polypropylene vial for direct LC–MS injection.

Nine target PFAS were confirmed and quantified: perfluorobutane sulfonate (PFBS), perfluorohexanoic acid (PFHxA), perfluorooctane sulfonate (PFOS), perfluorononanoic acid (PFNA), perfluorodecanoic acid (PFDeA), perfluoroundecanoic acid (PFUnA), perfluorododecanoic acid (PFDoA) and perfluorotridecanoic acid (PFTrDA). Data acquisition and processing were performed with Xcalibur software (Thermo Fisher Scientific). Compounds were identified in full scan (FS) mode by comparing retention times with calibration standards and confirmed by parallel reaction monitoring (PRM). Quantification was performed using response ratios between PFAS quantifier ions and the corresponding isotope-labelled internal standards; for analytes without a specific labelled internal standard, the internal standard with the closest retention time was used.

In addition to the targeted approach, raw HRMS files were processed using Compound Discoverer 3.1 (Thermo Scientific, USA) for non-target PFAS screening against the lists available in the NORMAN Suspect List Exchange database.

Supplementary Methods S3. Stable isotope analysis (detailed protocol)

Stable isotope analyses ($\delta^{13}C$ and $\delta^{15}N$) were performed on a subset of *Mustelus* specimens previously analyzed for organochlorine compounds; the number of samples selected for isotopic analysis (n = 25) was limited by available funding. Freeze-dried muscle samples from *M. mustelus* (n = 11) and *M. punctulatus* (n = 14) were weighed (0.2–0.3 mg) into tin capsules. Samples were analyzed by elemental analyzer–isotope ratio mass spectrometry (EA–IRMS) using a Thermo Finnigan FLASH EA 1112 Series CHN Analyzer coupled to a Thermo Finnigan Delta Plus XP isotope ratio mass spectrometer. Samples were combusted individually by flash combustion at approximately 1000°C to oxidize carbon to CO_2 and nitrogen to N_2.

Combustion gases passed through a quartz column with an oxidizing section packed with cobalt oxide to ensure complete oxidation, followed by a reducing section containing copper to remove excess oxygen and reduce nitrogen oxides to N_2. Water vapor was removed using an anhydrone trap, and the resulting gas mixture was separated by gas chromatography prior to IRMS. Reference standards were used for calibration and normalization through calibration curves. Additional reference pulses of CO_2 and N_2 were analyzed alongside samples. Helium (He) was used as carrier gas throughout. Reference gases were introduced into the IRMS through the CONFLO-III interface (Thermo Finnigan) controlled by ISODAT-NT software, which also recorded chromatographic peaks and supported data processing. Isotopic ratios were calibrated against international standards (IAEA-CH-6 and IAEA-CH-7) and reported in permill delta notation (δ ‰) relative to the international standard V-PDB for carbon and N_2 AIR for nitrogen [64]. Analytical error is calculated as cumulative error of duplicates and standards, resulting above 0.3 ‰ for all tested samples.

References numbered as in the main text:

28. Marsili, L.; Coppola, D.; Giannetti, M.; Casini, S.; Fossi, M.C.; van Wyk, J.H.; Sperone, E.; Tripepi, S.; Micarelli, P.; Rizzuto, S. Skin Biopsies as a Sensitive Non-Lethal Technique for the Ecotoxicological Studies of Great White Shark(Carcharodon Carcharias) Sampled in South Africa. Expert Opin Environ Biol 2016, 04, doi:10.4172/2325-9655.1000126.

62. Zeghnoun, A.; Pascal, M.; Fréry, N.; Sarter, H.; Falq, G.; Focant, J.F.; Eppe, G. Dealing with the Non-Detected and Non-Quantified Data. The Example of the Serum Dioxin Data in the French Dioxin and Incinerators Study.Pdf. Organohalogen Compounds 2007, 69, 2288–2291.

63. Mazzetti, M.; Marsili, L.; Valsecchi, S.; Roscioli, C.; Polesello, S.; Altemura, P.; Voliani, A.; Mancusi, C. First Investigation of Per-and Poly Fluoroalkylsubstances (PFAS) in Striped Dolphin Stenella Coeruleoalba Stranded along Tuscany Coast (North Western Mediterranean Sea). In Ninth International Symposium "Monitoring of Mediterranean Coastal Areas: Problems and Measurement Techniques"; Bonora, L., Carboni, D., De Vincenzi, M., Matteucci, G., Eds.; Firenze University Press: Florence, 2022; pp. 729–737 ISBN 979-12-215-0030-1.

64. Fry, Brian.; Brand, Willi.; Mersch, F.J.; Tholke, K.; Garritt, R. Automated Analysis System for Coupled .Delta.13C and .Delta.15N Measurements. Anal. Chem. 1992, 64, 288–291, doi:10.1021/ac00027a009.

3. Results

3.1. Biological parameters

Table S1. List of specimens collected in the Egadi Islands Marine Protected Area (MPAEI) during 2021–2023. The table reports sample ID, species, year of capture, sex, maturity stage and total length.

ID	Species	Sampled tissue	Year	Sex	Maturity Stage	TL (cm)	Weight (g)
MMU_13	Mustelus mustelus	Liver, Muscle	2021	Female	Juvenile	62	680
MMU_14	Mustelus mustelus	Liver, Muscle	2021	Female	Juvenile	65.5	950
MMU_15	Mustelus mustelus	Liver, Muscle	2021	Female	Juvenile	62	800
MMU_16	Mustelus mustelus	Liver, Muscle	2021	Female	Juvenile	56	550
MMU_17	Mustelus mustelus	Liver, Muscle	2023	Male	Juvenile	74	1500
MMU_18	Mustelus mustelus	Liver, Muscle	2023	Male	Adult	120	6200
MMU_19	Mustelus mustelus	Liver, Muscle	2023	Female	Juvenile	55	450
MMU_20	Mustelus mustelus	Liver, Muscle	2023	Female	Juvenile	63	800
MMU_21	Mustelus mustelus	Liver, Muscle	2023	Female	Adult	120	7500
MMU_22	Mustelus mustelus	Liver, Muscle	2023	Female	Adult	136	8500
MMU_23	Mustelus mustelus	Liver, Muscle	2023	Female	Juvenile	46	280
MMU_24	Mustelus mustelus	Liver, Muscle	2023	Female	Juvenile	80	2100
MMU_25	Mustelus mustelus	Liver, Muscle	2023	Female	Adult	150	14000
MMU_26	Mustelus mustelus	Liver, Muscle	2023	Female	Juvenile	44.5	196
MMU_27	Mustelus mustelus	Liver, Muscle	2023	Female	Adult	120	5500
MMU_28	Mustelus mustelus	Liver, Muscle	2023	Female	Adult	121	5600
MMU_29	Mustelus mustelus	Liver, Muscle	2023	Male	Adult	130	6000
MMU_30	Mustelus mustelus	Liver, Muscle	2023	Male	Maturing	97	3200
MMU_31	Mustelus mustelus	Liver, Muscle	2023	Female	Maturing	86	2200
MMU_32	Mustelus mustelus	Liver, Muscle	2023	Female	Maturing	85	1300
MMU_33	Mustelus mustelus	Liver, Muscle	2023	Male	Juvenile	61	700
MMU_34	Mustelus mustelus	Liver, Muscle	2023	Female	Juvenile	46	300
MMU_35	Mustelus mustelus	Liver, Muscle	2023	Female	Juvenile	50.3	355
MMU_36	Mustelus mustelus	Liver, Muscle	2023	Female	Adult	144	9000
MMU_37	Mustelus mustelus	Liver, Muscle	2023	Male	Maturing	88	2278
MMU_38	Mustelus mustelus	Liver, Muscle	2023	Female	Juvenile	74	1400
MMU_39	Mustelus mustelus	Liver, Muscle	2023	Female	Maturing	100	2740
MMU_40	Mustelus mustelus	Liver, Muscle	2023	Female	Juvenile	48	318
MPU_01	Mustelus punctulatus	Liver, Muscle	2022	Male	Juvenile	65	600
MPU_02	Mustelus punctulatus	Liver, Muscle	2022	Female	Maturing	80	1650

MPU_03	Mustelus punctulatus	Liver, Muscle	2022	Male	Juvenile	55	700
MPU_04	Mustelus punctulatus	Liver, Muscle	2022	Male	Juvenile	70	1000
MPU_05	Mustelus punctulatus	Liver, Muscle	2022	Female	Adult	115	5500
MPU_06	Mustelus punctulatus	Liver, Muscle	2023	Female	Adult	92	2000
MPU_07	Mustelus punctulatus	Liver, Muscle	2023	Female	Adult	100	3200
MPU_08	Mustelus punctulatus	Liver, Muscle	2023	Female	Maturing	86	2000
MPU_09	Mustelus punctulatus	Liver, Muscle	2023	Female	Adult	110	6300
MPU_10	Mustelus punctulatus	Liver, Muscle	2023	Female	Maturing	80	1300
MPU_11	Mustelus punctulatus	Liver, Muscle	2023	Male	Adult	94	2500
MPU_12	Mustelus punctulatus	Liver, Muscle	2023	Female	Maturing	87	2000
MPU_13	Mustelus punctulatus	Liver, Muscle	2023	Female	Adult	100	3000
MPU_14	Mustelus punctulatus	Liver, Muscle	2023	Male	Adult	100	2500
MPU_15	Mustelus punctulatus	Liver, Muscle	2023	Female	Maturing	88	1900
MPU_16	Mustelus punctulatus	Liver, Muscle	2023	Female	Maturing	84	1800
MPU_17	Mustelus punctulatus	Liver, Muscle	2023	Female	Maturing	80	1800
MPU_18	Mustelus punctulatus	Liver, Muscle	2023	Female	Maturing	75	1200
MPU_19	Mustelus punctulatus	Liver, Muscle	2023	Female	Maturing	73	1050
MPU_20	Mustelus punctulatus	Liver, Muscle	2023	Female	Maturing	75	1300
MPU_21	Mustelus punctulatus	Liver, Muscle	2023	Male	Maturing	75	1100
MPU_22	Mustelus punctulatus	Liver, Muscle	2023	Female	Maturing	85	2200
MPU_23	Mustelus punctulatus	Liver, Muscle	2023	Male	Maturing	75	1300
MPU_24	Mustelus punctulatus	Liver, Muscle	2023	Male	Adult	150	3000
MPU_25	Mustelus punctulatus	Liver, Muscle	2023	Female	Maturing	78	1200
MPU_26	Mustelus punctulatus	Liver, Muscle	2023	Female	Adult	152	16000
MPU_27	Mustelus punctulatus	Liver, Muscle	2023	Female	Maturing	86	2000
MPU_28	Mustelus punctulatus	Liver, Muscle	2023	Female	Maturing	83	1600
MPU_29	Mustelus punctulatus	Liver, Muscle	2023	Female	Adult	130	10000
MPU_30	Mustelus punctulatus	Liver, Muscle	2023	Female	Maturing	73	1160
MPU_31	Mustelus punctulatus	Liver, Muscle	2023	Female	Maturing	72.5	1020
MPU_32	Mustelus punctulatus	Liver, Muscle	2023	Female	Maturing	84	1800
MPU_33	Mustelus punctulatus	Liver, Muscle	2023	Female	Adult	130	7700
MPU_34	Mustelus punctulatus	Liver, Muscle	2023	Male	Adult	91	2300
MPU_35	Mustelus punctulatus	Liver, Muscle	2023	Male	Maturing	85	1800
MPU_36	Mustelus punctulatus	Liver, Muscle	2023	Male	Maturing	74.3	1200
MPU_37	Mustelus punctulatus	Liver, Muscle	2023	Female	Maturing	82	1600
MPU_38	Mustelus punctulatus	Liver, Muscle	2023	Female	Adult	130	7600
MPU_39	Mustelus punctulatus	Liver, Muscle	2023	Male	Maturing	76.7	1400
MPU_40	Mustelus punctulatus	Liver, Muscle	2023	Female	Adult	102	3700
MPU_41	Mustelus punctulatus	Liver, Muscle	2023	Male	Juvenile	57.5	605
MPU_42	Mustelus punctulatus	Liver, Muscle	2023	Female	Maturing	74	1300
MPU_43	Mustelus punctulatus	Liver, Muscle	2023	Female	Adult	120	6500

3.3. PFAS contamination levels in elasmobranch tissues

Table S2. Biometric and biological information for *Mustelus* sp. individuals analyzed for PFAS. ID: specimen code; TL: total length (cm); W: body weight (kg); F: female; M: male.

ID	Year	TL (cm)	W (kg)	Sex	Maturity stage
MMU_18M	2023	120	6.2	M	Adult
MMU_29M	2023	130	6	M	Adult
MPU_11M	2023	94	2.5	M	Adult
MPU_34M	2023	91	2.3	M	Adult
MMU_17M	2023	74	1.5	M	Juvenile
MMU_33M	2023	61	0.7	M	Juvenile
MPU_03M	2022	55	0.7	M	Juvenile
MPU_04M	2022	70	1	M	Juvenile
MMU_21M	2023	120	7.5	F	Adult
MMU_25M	2023	150	14	F	Adult
MPU_26M	2023	152	16	F	Adult
MPU_33M	2023	130	7.7	F	Adult
MPU_30M	2023	73	1.16	F	Maturing
MPU_31M	2023	72.5	1.02	F	Maturing
MMU_34M	2023	46	0.3	F	Juvenile
MMU_12M	2021	80	1.85	F	Juvenile

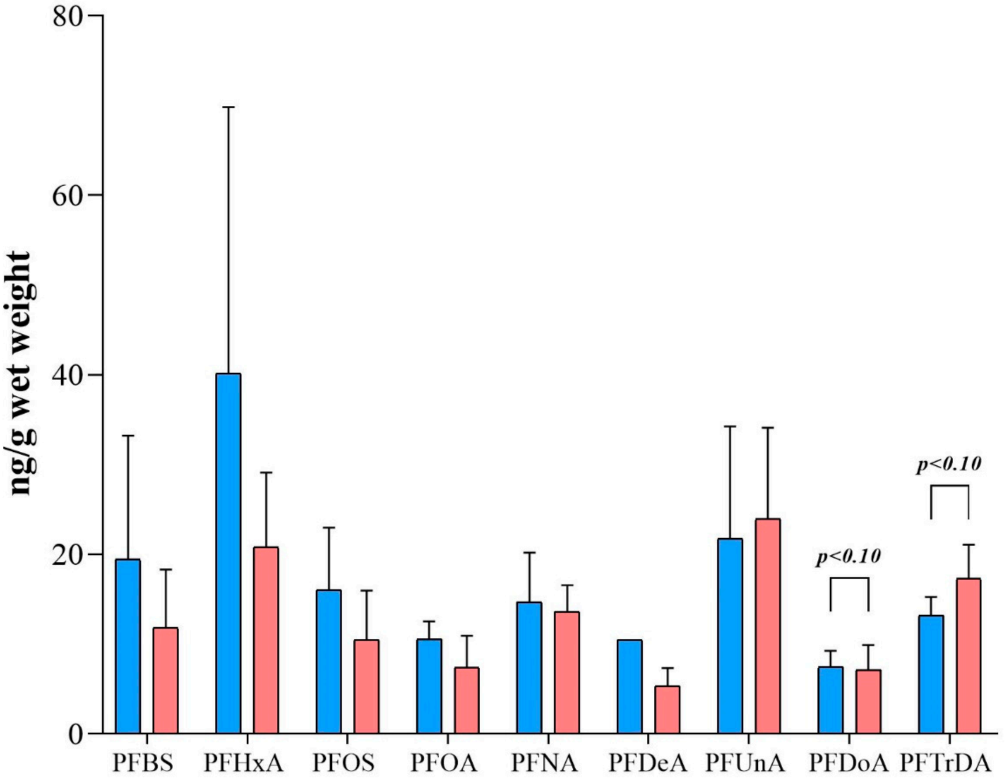

Figure S1. Mean PFAS concentrations (ng/g wet weight) in muscle tissue of *Mustelus* sp. (males, n = 8; females, n = 8). Bars show mean values and error bars indicate standard deviation.

3.4. Stable Isotopes analysis ($\delta^{13}C$ and $\delta^{15}N$)

Table S3. Stable isotope results for *M. mustelus* and *M. punctulatus*. The table includes sample ID, sex, total length (cm) and $\delta^{13}C$ and $\delta^{15}N$ values (‰).

Species	ID code	ID Isotopes	Sex	Length (cm)	$\delta^{15}N$ (‰)	$\delta^{13}C$ (‰)
M. mustelus	MMU_12M	MAS01	Female	80	7.14	-18.34
M. mustelus	MMU_13M	MAS03	Female	62	7.38	-18.12
M. mustelus	MMU_14M	MAS02	Female	65.5	7.12	-17.94
M. mustelus	MMU_15M	MAS04	Female	62	7.94	-18.05
M. mustelus	MMU_16M	MAS05	Female	56	7.18	-18.17
M. mustelus	MMU_17M	MAS13	Male	74	7.03	-18.18
M. mustelus	MMU_18M	MAS14	Male	120	7.33	-18.88
M. mustelus	MMU_19M	MAS22	Female	55	8.37	-17.35
M. mustelus	MMU_20M	MAS23	Female	63	8.57	-17.09
M. mustelus	MMU_25M	MAS33	Female	150	8.26	-18.16
M. mustelus	MMU_27M	MAS38	Female	120	8.39	-18.95
M. punctulatus	MPU_01M	MAS09	Male	65	6.81	-18.96
M. punctulatus	MPU_02M	MAS07	Female	80	7.15	-17.23
M. punctulatus	MPU_03M	MAS10	Male	55	7.07	-18.25
M. punctulatus	MPU_04M	MAS08	Male	70	8.37	-17.24
M. punctulatus	MPU_05M	MAS06	Female	115	7.77	-18.57
M. punctulatus	MPU_06M	MAS11	Female	92	6.87	-18.79
M. punctulatus	MPU_09M	MAS12	Female	110	7.32	-18.40
M. punctulatus	MPU_11M	MAS15	Male	94	7.46	-19.14
M. punctulatus	MPU_14M	MAS16	Male	100	7.59	-18.63
M. punctulatus	MPU_19M	MAS19	Female	73	6.71	-25.21
M. punctulatus	MPU_21M	MAS20	Male	75	8.57	-19.24
M. punctulatus	MPU_22M	MAS21	Female	85	8.55	-18.97
M. punctulatus	MPU_31M	MAS36	Female	72.5	8.04	-24.07
M. punctulatus	MPU_32M	MAS37	Female	84	8.15	-22.48

3.4.1. Comparative interpretation of OCs and Stable Isotopes

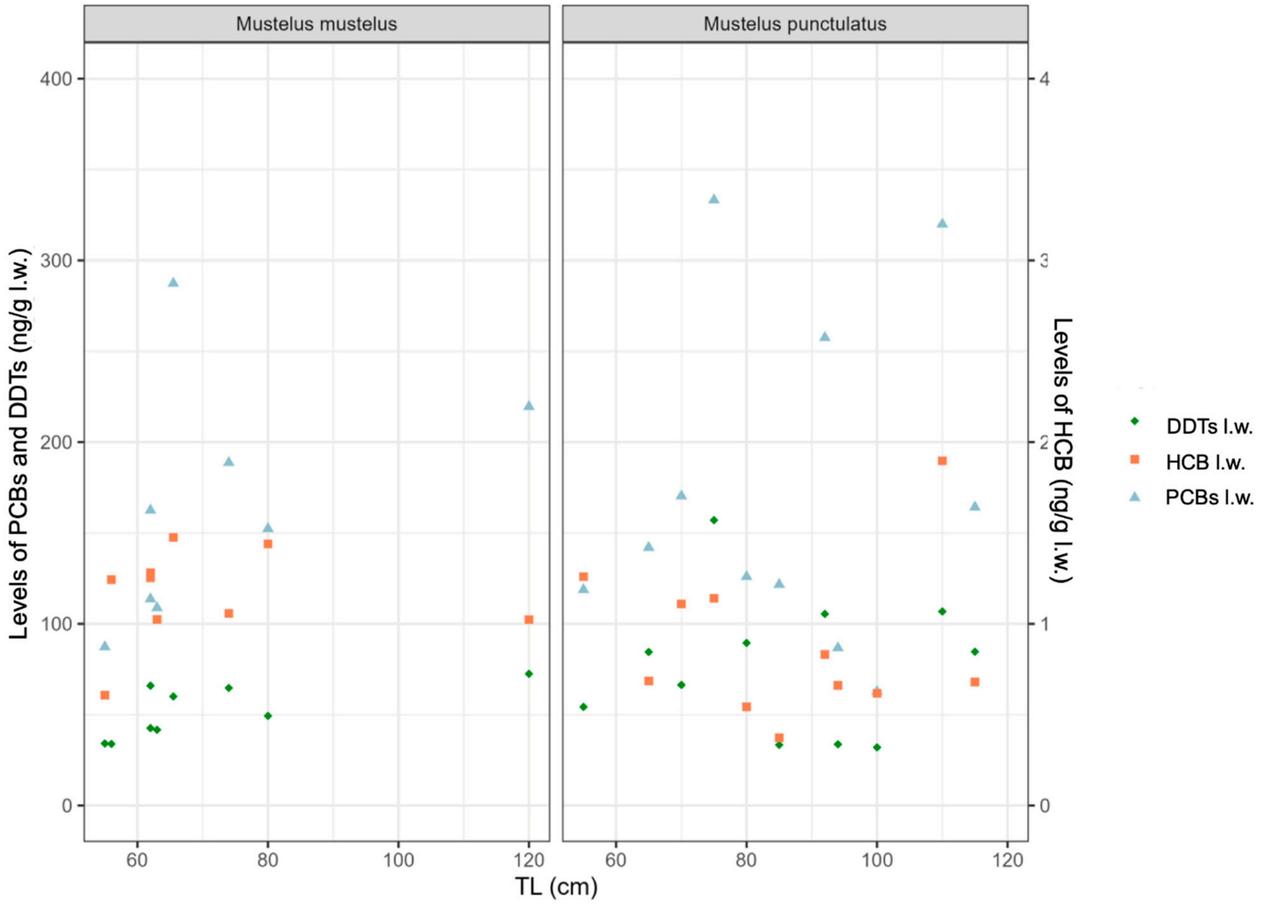

Figure S2. Biometric and biological information for *Mustelus* sp. individuals analyzed for PFAS. ID: specimen code; TL: total length (cm); W: body weight (kg); F: female; M: male.

3.4.1.1. Levels of OCs in relation to $\delta^{13}C$ and $\delta^{15}N$

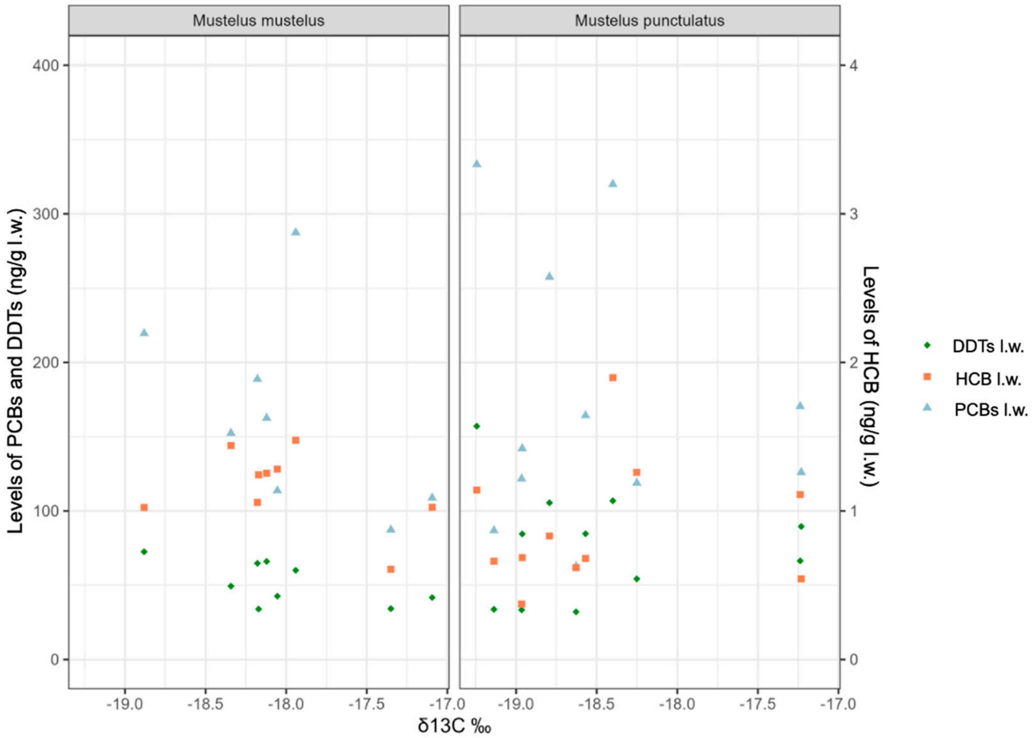

Figure S3. Relationship between $\delta^{13}C$ (‰) and OC concentrations (ng/g lipid weight) in M. mustelus and M. punctulatus. Concentrations of HCB, ΣDDTs and ΣPCBs are shown as a function of $\delta^{13}C$.

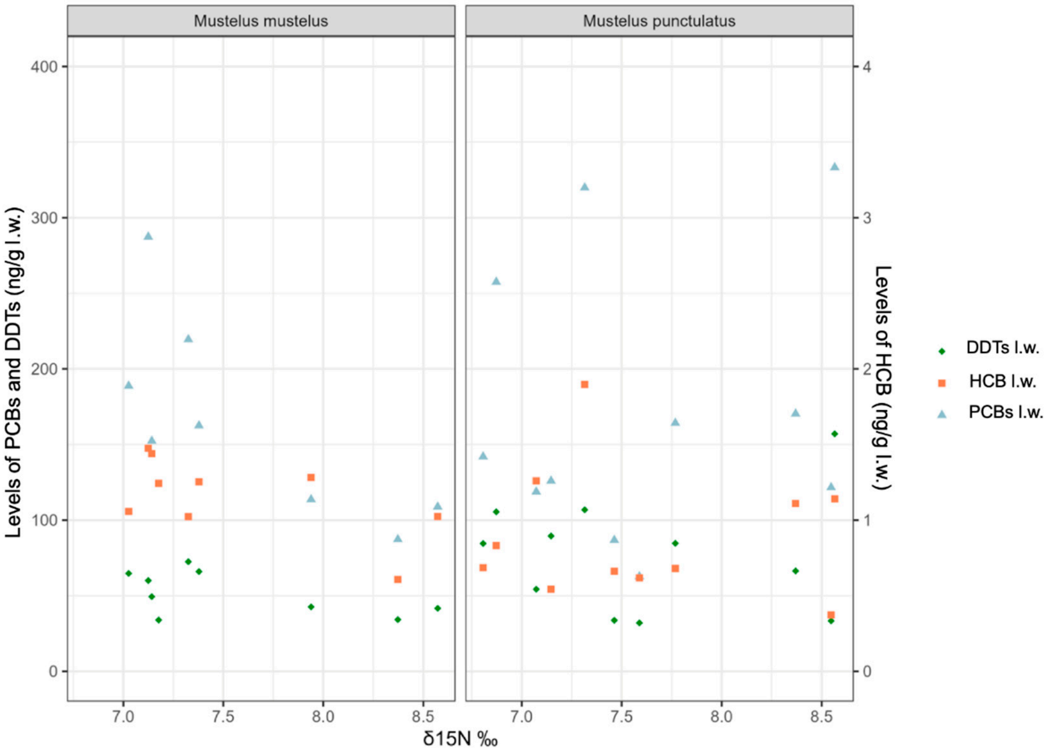

Figure S4. Relationship between $\delta^{15}N$ (‰) and OC concentrations (ng/g lipid weight) in *M. mustelus* and *M. punctulatus*. Concentrations of HCB, ΣDDTs and ΣPCBs are shown as a function of $\delta^{15}N$.

3.4.2. PFAS concentrations in relation to δ13C and δ15N

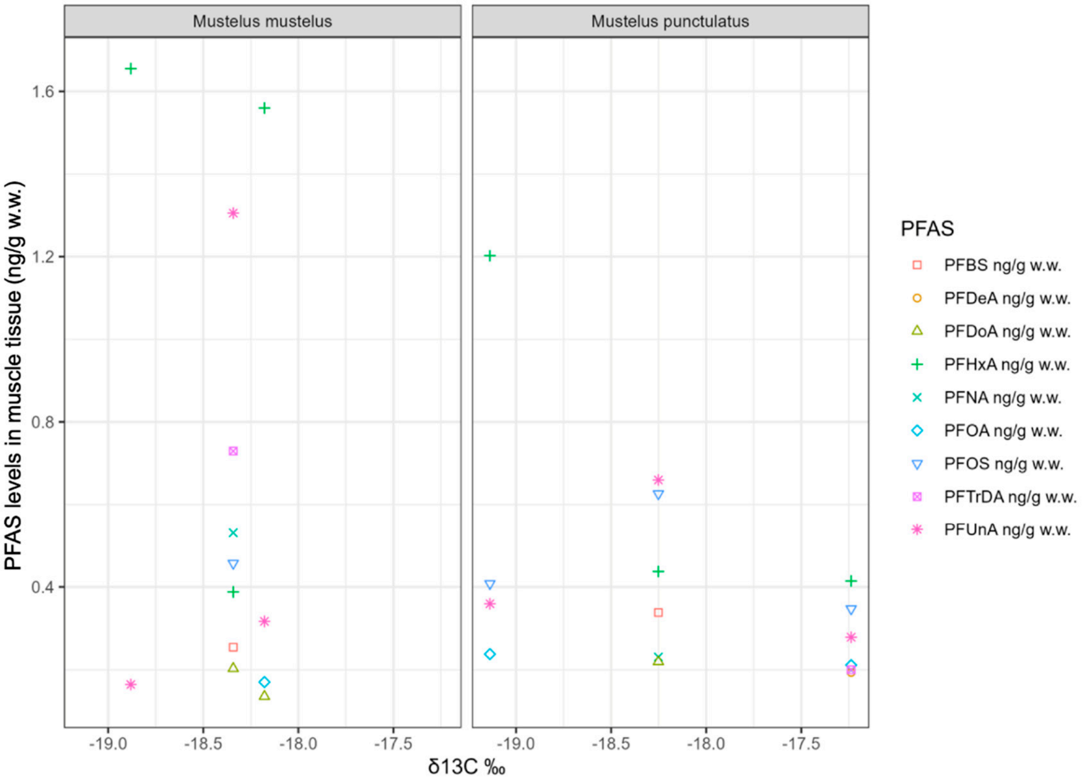

Figure S5. Relationship between $\delta^{13}C$ (‰) and PFAS concentrations (ng/g wet weight) in *M. mustelus* and *M. punctulatus*.

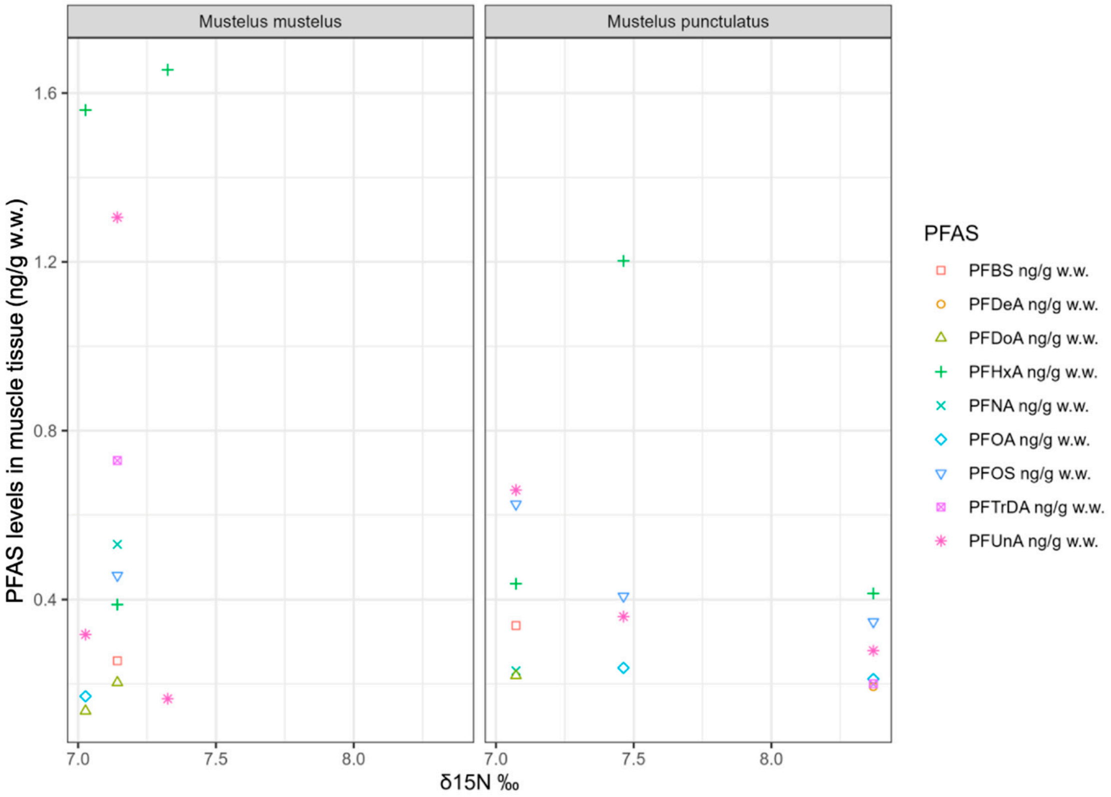

Figure S6. Relationship between $\delta^{15}N$ (‰) and PFAS concentrations (ng/g wet weight) in *M. mustelus* and *M. punctulatus*.

3.5.2. PFAS evaluation in exposure risk assessment

Table S4. PFAS concentrations measured in muscle samples for PFOS, PFOA and PFNA, together with their sum, reported for comparison with EU Regulation 2023/9155.

ID	PFOS	PFOA	PFNA	Σ (PFOS, PFOA, PFNA)
MMU_21M	0.077	0.075	0.276	0.428
MPU_26M	0.056	< LOQ	0.043	0.100
MMU_25M	< LOQ	0.025	0.056	0.081
MPU_30M	0.160	0.088	0.084	0.332
MPU_31M	0.143	0.077	0.185	0.405
MPU_33M	0.025	0.054	< LOQ	0.080
MPU_34M	0.114	< LOQ	0.276	0.390
MMU_29M	< LOQ	0.059	< LOQ	0.059
MMU_33M	0.424	< LOQ	0.821	1.244
MMU_34M	0.099	0.054	0.209	0.362
MMU_17M	< LOQ	0.171	< LOQ	0.171
MMU_18M	< LOQ	< LOQ	< LOQ	< LOQ
MPU_11M	0.408	0.238	< LOQ	0.646
MMU_12M	0.457	< LOQ	0.531	0.987
MPU_03M	0.626	< LOQ	0.231	0.857
MPU_04M	0.347	0.211	0.200	0.758
